# Retreatment with Bendamustine-Bortezomib-Dexamethasone in a Patient with Relapsed/Refractory Multiple Myeloma

**DOI:** 10.1155/2016/6745286

**Published:** 2016-10-27

**Authors:** Claudio Cerchione, Davide Nappi, Maria Di Perna, Irene Zacheo, Anna Emanuele Pareto, Marco Picardi, Lucio Catalano, Fabrizio Pane

**Affiliations:** Hematology, University Federico II, Via Pansini 5, 80131 Napoli, Italy

## Abstract

The clinical management of relapsed/refractory multiple myeloma and the correct choice of the most suitable therapy in heavily pretreated and fragile patients are tough clinical issues for clinicians. In advanced phases of disease, the choice of available therapies becomes very poor, and the retreatment with previously adopted and effective therapy, although unpredictable, could be an effective option. In this report, we describe the clinical history of a patient, previously treated with 9 lines of therapy, refractory to bortezomib and IMIDs, for whom the retreatment with bendamustine resulted in a stable disease with good quality of life.

## 1. Introduction

In advanced multiple myeloma, the choice of the treatment can be difficult, as therapeutic options decrease over time. Both new combinations of previously used drugs and retreatment with a previously adopted and effective therapy can be taken into consideration in patients showing persistent chemosensitivity. In this report, we describe the case of a heavily pretreated patient, refractory to bortezomib and IMIDs, with clinical benefit after retreatment with bendamustine.

## 2. Case Presentation

In June 2009, this male patient was 67 years old and was diagnosed with IgG *λ* stage IIIA multiple myeloma (MM).* FISH analysis was performed at diagnosis, and it showed negativity for the most frequent alterations (t(11; 14), t(4; 14), del13q, and del17p)*. First-line therapy was 7 cycles of thalidomide-dexamethasone (TD), followed by radiotherapy on T2. In March 2010 progressive* bone* disease was detected by MRI of the spine showing multiple cervical and dorsal osteolytic lesions. Thus, second line of bortezomib-desametasone (VD), together with zoledronic acid, was performed for 5 cycles, obtaining a partial response. A first ASCT, preceded by thiotepa/melphalan conditioning regimen, was performed in December 2010 leading to a partial response. After a period with stable clinical conditions, in April 2011, disease progression* was documented by the increase of the serum monoclonal component (sMC)*: the patient was treated with 4 courses of lenalidomide-dexamethasone (RD), but the disease progressed. Therefore, a combination of melphalan-lenalidomide-dexamethasone (MRD) was performed for 3 cycles in September 2011, again followed by disease progression,* determined by sMC increase*. At the same time, PET/CT performed for neck pain revealed multiple osteolytic lesions: the most dangerous (C2) was treated with tomotherapy (40 Gy total). Thus, 2 cycles of cyclophosphamide-doxorubicin-dexamethasone (CED) regimen were attempted (1), but the disease was still refractory. Hence, a bendamustine-bortezomib-dexamethasone (BVD) regimen was administered (bendamustine 90 mg/sqm at days 1 and 2, bortezomib 1.3 mg/sqm at days 1, 4, 8, and 11, dexamethasone 20 mg at days 1, 2, 4, 5, 8, 9, 11, and 12, and pegfilgrastim 6 mg at day + 4) (2, 3, and 4) for 6 cycles, resulting in a partial response, followed by a second ASCT, preceded by thiotepa/melphalan conditioning regimen. In February* 2014, a further sMC increase suggested disease progression, and* the patient was treated with bortezomib-lenalidomide-dexamethasone (VRD) for 6 cycles with the result of progressive disease. In November 2014, for disease progression confirmed also by PET/CT scan (Table 1), even considering cardiovascular comorbidities, BVD-retreatment was chosen as tenth line. The patient switched to a stable disease status and clinical conditions were relatively fit for more than one year. The treatment was well tolerated: the only toxicities were grade 2 anemia and grade 3 thrombocytopenia, while severe neutropenia was effectively prevented with pegfilgrastim prophylaxis (6 mg at day + 4 of every courses). No extrahematological side effects were revealed.

Due to* further sMC increase*, in December 2015, 4 courses of pomalidomide-dexamethasone were attempted, in a palliative intent, but the patient died in July 2016.

## 3. Discussion

After the advent of proteasome inhibitors, international guidelines agree on first-line treatment strategy for ASCT-eligible and noneligible patients [1–3]. However, selecting and managing the correct therapy for a patient with rrMM it is still a tough task for the hematologist, as, after many relapses, available therapeutic options are scanty. A commonly adopted strategy consists in retreating the patient with the same molecules used previously, choosing those which showed the best response or considering new drug combinations,* even if in previous administrations single drugs showed to be ineffective* [4–9].

This strategy seems particularly successful in patients who show persistent chemosensitivity, as in our case, who obtained an overall survival longer than 7 years, which can be considered as an impressive result in a 67-year-old patient affected by MM.

Bendamustine is a well-tolerated agent with a double mechanism of action, alkylating and antimetabolite, with proved effectiveness in treatment of relapsed/refractory [10, 11] and newly diagnosed multiple myeloma [12, 13] and in a relapsing/refractory setting [14–19]. In rrMM it can be used as single agent combined to dexamethasone, but a synergistic effect has been demonstrated when associated with bortezomib.

Bendamustine showed significant efficacy also in a selected setting of patients, such as those who became refractory to bortezomib and IMIDs or multirelapsed after single or double ASCT, demonstrating also an effective opportunity as a bridge to ASCT [10]. To the best of our knowledge, BVD-retreatment for relapsing/refractory MM is still not consolidated, but, as in our case, it could be considered an effective choice in heavily pretreated patients without significant therapeutic options, in a context of a well-tolerated palliative treatment with good quality of life.

## Figures and Tables

**Table 1:** Patient's history.

Line	Regimen	Cycle (n°)	Responses
1	Thalidomide-dexamethasone + RT	7	Progressive disease
2	Bortezomib-dexamethasone	5	Partial response
3	First auto-BMT (thiotepa-melphalan)	/	Stable disease
4	Lenalidomide-dexamethasone	4	Progressive disease
5	Melphalan-lenalidomide-dexamethasone	3	Progressive disease
6	Doxorubicin-cyclophosphamide-dexamethasone	2	Progressive disease
7	Bendamustine-bortezomib-dexamethasone	6	Partial response
8	Second auto-BMT (thiotepa-melphalan)	/	Stable disease
9	Bortezomib-lenalidomide-dexamethasone	6	Progressive disease
10	Bendamustine-bortezomib-dexamethasone	7	Stable disease
11	Pomalidomide-dexamethasone	4	Progressive disease
